# Investigation of chronic diseases in patients with inflammatory bowel disease: A hospital-based case-control study

**DOI:** 10.22088/cjim.13.2.7

**Published:** 2022

**Authors:** Ali Zabihi, Mehrdad Kashifard, Seyedeh Roghayeh Jafarian Amiri, Mahdi Sepidarkish, Valiollah Padehban, Mojtaba Qanbari Qalehsari

**Affiliations:** 1 Social Determinants of Health Research Center, Health Research Institute, Babol University of Medical Sciences, Babol, Iran; 2 Department of Internal Medicine, School of Medicine, Rouhani Hospital, Babol University of Medical Sciences, Babol, Iran; 3 Nursing Care Research Center, Health Research Institute, Nursing & Midwifery School, Babol University of Medical Sciences, Babol, Iran; 4 Department of Epidemiology and Biostatistics, School of Public Health, Infertility and Reproductive Health Research Center, Health Research Institute, Babol University of Medical Sciences, Babol, Iran; 5 Student research committee, School of Nursing & Midwifery, Golestan University of Medical Sciences, Gorgan, Iran

**Keywords:** Case control study, Chronic disease, Inflammatory bowel disease

## Abstract

**Background:**

Inflammatory bowel disease (IBD) is a broad term that refers to a group of chronic inflammatory disorders that have an unknown origin and might be associated with other diseases. The aim of this study was to determine the frequency of chronic diseases in patients with IBD.

**Methods:**

In this case-control study, 280 patients with IBD were compared with 280 healthy individuals, frequency-matched by age, sex, place of residence and marital status. Random sampling was performed in patients that referred to the internal medicine and gastroenterology wards of hospitals affiliated to Babol University of Medical Sciences. Data collection tools included a demographic questionnaire and a checklist for chronic diseases, which were completed through interviews with the case and control groups.

**Results:**

Two hundred and twenty-nine (81.78%) patients with IBD had at least one chronic disease. Patients with IBD were at increased risks of rheumatoid arthritis (OR= 4.48, 95%CI: 1.48, 13.54, P= 0.008), eye diseases (OR= 3.49, 95%CI: 1.68, 7.28, P= 0.001), liver diseases (OR= 2.74, 95%CI: 1.40, 5.34, P= 0.003 ), anemia (OR = 2.53, 95%CI: 1.56, 4.13, P= 0.000), depression (OR= 2.43, 95%CI: 1.58, 3.74, P= 0.000), skin diseases (OR= 2.36, 95%CI: 1.18, 4.74, P= 0.015) and hypertension (OR= 1.77, 95%CI: 1.06, 2.95, P= 0.028).

**Conclusion:**

The frequency of chronic diseases associated with IBD has been high, therefore, physicians and health care professionals should consider the possibility of other chronic diseases when dealing with IBD patients.

Inflammatory bowel disease (IBD) is a general term used for a group of chronic diseases of the gastrointestinal tract that the most common form of which are ulcerative colitis (UC) and Crohn's Disease (CD) (1). Due to the autoimmune nature of the disease, many cases are associated with extra-intestinal complications of IBD or other chronic diseases. Patients with IBD may be simultaneously diagnosed with other diseases such as blood, bone, heart and liver involvement. Some of these diseases may develop as a result of inflammation of the intestine or independently of IBD (2). Overall, about 30% of extra-intestinal disorders are detected before IBD is diagnosed (3). Patients with IBD may be simultaneously diagnosed with other diseases. Some of these diseases may develop as a result of inflammation of the intestine or independently of IBD (4). Numerous studies have investigated the extra-intestinal complications of patients with IBD, often assessing only one or a small number of extra-intestinal complications (3). However, there are not any comprehensive studies that assess chronic diseases in IBD patients.

Early diagnosis and screening for diseases associated with IBD can help prevent disease progression, treatment, and reduce the expenses for health system and community. The aim of this study was to investigate the chronic diseases in patients with IBD.

## Methods

This case-control study was conducted from January to July 2020 on 280 patients with IBD in the case group and 280 healthy and homogeneous individuals in the control group after being approved by the Research Ethics Committee of Babol University of Medical Sciences under the ethical approval code: IR .MUBABOL.HRI.REC.1398.159. 

Based on the Cochran table, assuming a statistical population of 1,000 (5), the sample size is estimated at 278. Sampling was performed using simple random sampling among patients who referred to internal medicine, gastroenterology and endoscopy wards as well as outpatient clinics of hospitals affiliated to Babol University of Medical Sciences. Diagnosis of the disease in the case group was confirmed by internal medicine and gastroenterologists using ECCO-ESGAR guidelines (6). 

The control group frequency-matched the case group in terms of age, sex, place of residence and marital status. The participants in this group were individuals who had no history of IBD and other gastrointestinal diseases in their medical records and on initial examination by a physician and were selected from the outpatient clinics of hospitals affiliated to Babol University of Medical Sciences. 

The presence or absence of chronic diseases in participants was determined by examining their medical records and by asking them if the doctor has ever told them that they have a specific disease. If a participant reported that he or she had a specific health problem, his or her medical record was reviewed, and if he or she did not have a medical condition, he or she was considered healthy.

Chronic diseases including diabetes, hypertension, cardiovascular disease, chronic obstructive pulmonary disease, autoimmune diseases of the nervous system, depression, liver disease, anemia, arthritis, skin and eye diseases, were recorded through the patients’ self-report and were confirmed via their medical records. Data collection tools in this study included a demographic information questionnaire and a checklist for chronic diseases. Logistic regression was to investigate the association between chronic diseases and IBD. The final multivariate models were adjusted for the following risk factors: age, gender, body mass index, smoking, OCP consumption and NSAIDs use. Variable selection was down based on the prior knowledge. 

## Results

About 60% of IBD patients had normal weight (BMI: 18.5-24.9). There was no significant difference between the case and the control groups in terms of non-steroidal anti-inflammatory drugs and oral contraceptive pills (table 1). Regarding the chronic diseases, only 51 (18.21%) patients with IBD did not have chronic diseases, while in the control group 105 (37.5) individuals did not have chronic diseases (P=0.000). Among the patients with IBD, 16.1% were hypertensive. The patients with IBD were 1.7 times more likely to develop hypertension (aOR = 1.77 95%CI: 1.06, 2.95) than the patients in the control group.

Thirty three (11.8%) of patients with IBD had chronic liver disease. After adjusting confounder variables, patients with IBD were 2.74 times more likely to develop chronic liver disease than non-IBD patients (aOR=2.74, 95%CI: 1.40, 5.34, P=0.003). Seventy four (28.2%) patients with IBD had depression. Patients with IBD were 2.43 times more likely to develop depression than non-IBD patients (aOR= 2.43, 95%CI: 1.58, 3.74, P=0.000). Eighteen (6.4%) of patients with IBD had rheumatoid arthritis. 

Patients with IBD were 4.48 times more likely to develop rheumatoid arthritis than non-IBD patients (aOR= 4.48, 95%CI: 1.48, 13.54, P=0.000). Sixty-one (21.8%) patients with IBD had anemia. Patients with IBD were 2.53 times more likely to develop anemia than non-IBD patients (aOR= 2.53, 95%CI: 1.56, 4.13, P=0.000). Among the patients with IBD, 32(11.4%) had eye diseases. patients with IBD were 3.49 times more likely to develop eye disease than non-IBD patients (aOR= 3.49, 95%CI: 1.68, 7.28, P=0.001). Twenty seven (9.6%) of IBD patients had skin diseases. IBD patients were 2.36 times more likely to develop skin diseases than non-IBD patients (aOR= 2.36, 95%CI: 1.18, 4.74, P=0.015) (table 2(.

**Table 1:** Background factors in IBD cases and controls

	Cases (n= 280)	Controls (n= 280)	P-Value^‡^
Age categories (years) †			
**< 20**	34 (12.14)	30 (10.71)	0.995
**20-29**	37 (13.21)	37 (13.21)	
**30-39**	56 (20)	55 (19.64)	
**40-49**	47 (16.79)	47 (16.79)	
**50-59**	58 (20.71)	59 (21.07)	
**≥ 60**	48 (17.14)	52 (18.57)	
BMI (kg/m2)			
**> 18.5**	48 (17.14)	54 (19.29)	0.374
**18.5-24.9**	167 (59.64)	154 (55)	
**25-29.9**	49 (17.50)	61 (21.79)	
**≥ 30**	16 (5.71)	11 (3.93)	
Gender			
**Male**	148 (52.86)	148 (52.86)	0.999
**Female**	132 (47.14)	132 (47.14)	
Residence			
**Urban**	145 (51.79)	166 (59.29)	0.074
**Rural**	135 (48.21)	114 (40.71)	
**Smoking**	38 (13.57)	51 (18.21)	0.133
**NSAIDs usage**	233 (83.21)	226 (80.71)	0.442
**OCP consumption**	58 (20.71)	54 (19.29)	0.883

BMI: body mass index; NSAIDs: Non-steroidal anti-inflammatory drugs; OCP: oral contraceptive pill

†: Values given as number (percentage)

‡ Variables compared with chi-square test.

**Table 2:** Multivariate Regression Analysis of Predictors of chronic diseases in IBD Cases

Disease	Case Frequency (%)	Control Frequency (%)	Crude OR (95% CI)	P-Value	Adjusted OR (95% CI) †	P-Value*
**Diabetes Mellitus**	44(15.7)	49(17.5)	0.87 (0.56, 1.37)	0.570	0.86 (0.55, 1.35)	0.537
**Hypertension**	45(16.1)	27(9.6)	1.79 (1.07, 2.98)	0.024	1.77 (1.06, 2.95)	0.028
**Liver diseases**	33(11.8)	13(4.6)	2.74 (1.41, 5.33)	0.003	2.74 (1.40, 5.34)	0.003
**COPD**	12(4.3)	13(4.6)	0.91 (0.41, 2.05)	0.838	0.93 (0.41, 2.09)	0.868
**Depression**	79(28.2)	40(14.3)	2.35 (1.54, 3.60)	<0.001	2.43 (1.58, 3.74)	< 0.001
**Rheumatoid arthritis**	18(6.4)	4(1.4)	4.74 (1.58, 14.19)	0.005	4.48 (1.48, 13.54)	0.008
**Neoplasms**	4(1.4)	3(1.1)	1.33 (0.29, 6.03)	0.705	1.24 (0.27, 5.66)	0.779
**Anaemia**	61(21.8)	28(10)	2.98 (1.54, 4.06)	<0.001	2.53 (1.56, 4.13)	< 0.001
**Chronic kidney disease**	19(6.8)	22(7.9)	0.85 (0.45, 1.61)	0.627	0.84 (0.44, 1.61)	0.618
**Osteoporosis**	23(8.2)	13(4.6)	1.83 (0.91, 3.70)	0.089	1.90 (0.94, 3.87)	0.074
**Eye diseases**	32(11.4)	10(3.6)	3.48 (1.67, 7.23)	0.001	3.49 (1.68, 7.28)	0.001
**Skin diseases**	27(9.6)	13(4.6)	2.19 (1.10, 4.34)	0.024	2.36 (1.18, 4.74)	0.015

†Odds ratio estimated directly from Multiple Logistic regression. The final multivariate models were adjusted for the following risk factors: age, gender, body mass index, smoking, OCP consumption and NSAIDs usage.

*Significant at Pvalue <0.05.

## Discussion

In this study, at least 81% of the patients with IBD had at least one or more chronic diseases. The high incidence of chronic diseases in this study may be due to the inclusion of and the focus on a wide range of chronic diseases, especially diseases such as hypertension, diabetes and depression. Multivariate regression analysis revealed that the association between IBD and chronic diseases including hypertension, liver disease, depression, rheumatoid arthritis, anemia, eye and skin diseases was significant.

**Rheumatoid Arthritis and IBD: **the patients with IBD were 4.48 times more likely to develop rheumatoid arthritis than non-IBD people. In a study by Min Bae et al., IBD patients were at 3.47 times greater risk of developing rheumatoid arthritis compared to non-IBD people (7). As in the present study, in other studies, rheumatoid arthritis has the most common association with IBD (8).

**Eye diseases and IBD: **In this study, IBD patients were 3.49 times more likely to develop eye diseases than non-IBD patients. Anterior uveitis/ iridocyclitis are the most common ocular complications of IBD (9). Treatment of IBD-associated ocular inflammation can range from corticosteroids to steroid-sparing immunosuppression such as azathioprine or methotrexate (10). 

**Liver diseases and IBD: **Patients with IBD were 2.74 times more likely to develop liver disease than non-IBD patients. Principi et al. in their study showed that younger patients with IBD were more likely to develop liver disease, although there was generally no significant association between IBD and liver disease (11). Silva et al. showed that 19.6% of patients with IBD had hepatic manifestations, of which 56.7% were identified after being diagnosed with IBD.

**Anemia and IBD: **Anemia is one of the most common extra-intestinal manifestations of IBD, which affects the progression and length of hospitalization of IBD patients. Factors influencing the incidence of anemia in these patients include iron loss due to gastrointestinal bleeding, vitamin B12 mal-absorption due to ileal involvement, and folate deficiency due to sulfasalazine consumption. Hemolytic anemia may also be seen in some IBD patients (12).In the present study, IBD patients were 2.53 times more likely to develop anemia than non-IBD patients. Among the types of anemia caused by IBD, iron deficiency anemia due to blood loss is more common. 

**Depression and IBD: **Chronic diseases like IBD expose people to mental illness. In the present study, IBD patients were 2.43 times more likely to develop depression than non-IBD patients. Bernstein et al. showed a significant increase in mental health problems in IBD patients, in a way that IBD patients were 1.58 times more likely to develop depression compared to non-IBD patients (13). Kochar et al. also showed that depression is highly prevalent in IBD patients, especially in the active phase of the disease. On the other hand, there is a two-way relationship between IBD and depression (14).

**Skin diseases and IBD: **In the present study, IBD patients were at 2.36 times greater risk of developing skin diseases compared to non-IBD patients. In the study of Jansen et al., 20% of IBD patients had skin problems and erythema nodosum was the most common skin disease associated with IBD (15). Young women with a family history of IBD are at greater risk for skin diseases (16).

**Hypertension and IBD: **Patients with IBD are 1.77 times more likely to develop hypertension than non-IBD patients. The study of Radovan Prijić et al. showed that patients with longer-term IBD had higher serum cholesterol, HDL, and ALx (Augmentation index). These patients also had higher PWV (pulse wave velocity) than patients with controlled blood pressure (17). Bigeh et al. (2019) stated that inflammatory diseases are associated with an increased risk of cardiovascular diseases including hypertension. Medications used to treat IBD such as steroids, can increase this risk. (18). 

In conclusion most patients with IBD had at least one other chronic disease that put them at higher risk for rheumatoid arthritis, eye disease, liver disease, anemia, depression, skin diseases, and high blood pressure, respectively. Physicians and health care givers should consider these diseases when dealing with patients with IBD to identify other chronic diseases associated with IBD and prevent the progression of the disease.
